# An infant with IVIG-resistant Kawasaki disease requiring repeated glucocorticoids therapy: A case report and literature review

**DOI:** 10.1097/MD.0000000000042572

**Published:** 2025-05-23

**Authors:** Yifan Ren

**Affiliations:** a Department of Pediatrics, Shaoxing Keqiao Women and Children’s Hospital, Shaoxing, PR China.

**Keywords:** case report, glucocorticoids, infant, IVIG-resistant Kawasaki disease, Kawasaki disease

## Abstract

**Rationale:**

Kawasaki disease (KD) is an acute systemic vasculitis predominantly affecting children, characterized by fever, chapped lips, strawberry tongue, conjunctival hyperemia, rash, and cervical lymphadenopathy. The most severe complication associated with KD is the development of coronary artery abnormalities.

**Patient concerns:**

A 1-year and 2-month-old girl was admitted to the hospital with persistent fever, rash, and elevated inflammatory markers.

**Diagnoses:**

Despite 36 hours of intravenous immunoglobulin (IVIG) therapy, she remained febrile and was classified as IVIG-resistant.

**Interventions:**

Methylprednisolone was administered for 13 days; however, tapering to a single daily dose resulted in symptom recurrence.

**Outcomes:**

A subsequent course of intravenous methylprednisolone, administered over 42 days, resolved her symptoms, with follow-up evaluations demonstrating normal echocardiograms and electrocardiograms. The duration of methylprednisolone treatment exceeded standard guideline recommendations for KD; however, the patient achieved full recovery without adverse effects.

**Lessons:**

This case highlights the potential efficacy of glucocorticoids as a treatment option for IVIG-resistant KD and emphasizes the necessity for further research into their role as first-line therapy, particularly in patients with IVIG intolerance.

## 1. Introduction

Following initial treatment, 10% to 20% of patients with Kawasaki disease (KD) experience recurrent fever, a condition defined as IVIG resistance.^[1]^ Retreatment with IVIG remains the most widely accepted therapy for IVIG-resistant KD, largely due to the lack of standardized treatment protocols in this field. Rescue therapies, including corticosteroids and infliximab, are also commonly utilized in clinical practice.^[2]^

In this case report, high-dose methylprednisolone was administered as a treatment for IVIG-resistant KD. However, during the tapering phase of the methylprednisolone dose, the patient experienced a recurrence of fever. To manage this recurrence, intravenous methylprednisolone was reinitiated and gradually tapered over time. This treatment regimen was maintained for a total of 42 days, resulting in significant clinical improvement in the patient. This case may offer valuable insights into the potential role of glucocorticoid therapy in the management of IVIG-resistant KD.

## 2. Case presentation

The patient was a 1-year-and-2-month-old girl weighing 9.5 kilograms (kg) who was admitted to the hospital with a 3-day history of fever and a 1-day history of rash. She exhibited no respiratory symptoms, such as rhinorrhea, nasal congestion, or cough, nor any urinary symptoms, such as dysuria or discomfort during urination. The initial blood test results revealed elevated C-reactive protein (CRP) levels and an increased white blood cell (WBC) count with neutrophilic predominance (Table 1).

**Table 1:** Laboratory investigation.

Test	Results of the first blood test	Results of the second blood test	Results of the third blood test	Reference range
WBC	16.2	10.9	16.3	5.5–13.6 × 10^9^/L
N%	79.4	79.4	76.1	40%–75%
L%	16.2	14.8	17.9	40%–60%
Hb	108	105	108	110–150 g/L
PLT	225	210	413	100–300 × 10^9^/L
CRP	60	81.8	5.7	0–8 mg/L
UA		181		155–357 μmol/L
BUN		2.77		2.30–6.70 mmol/L
SCR		25		13–33 μmol/L
ALT		375	55	8–42 U/L
AST		327	23	22–59 U/L
Total protein		57.6	64.4	58–76 g/L
Total bilirubin		40.20	3.6	3.42–20.50 μmol/L
Direct bilirubin		33.7	2.1	0–6.84 μmol/L
Sodium		132.9		135–145 mmol/L
B/A ratio		1.70	0.79	1.30–3.50
Albumin		36.3	28.5	39–54 g/L
PCT		1.71		0–0.5 ng/mL
ESR		74.8		0–20 mm/h

Abbreviations: ALT = serum alanine aminotransferase, AST = aspartate aminotransferase, B/A ratio = bilirubin-to-albumin ratio, BUN = blood urea nitrogen, ESR = erythrocyte sedimentation rate, Hb = hemoglobin, L% = percentage of lymphocytes, N% = percentage of neutrophils, PCT = procalcitonin, PLT = platelets, SCR = serum creatinine, UA = uric acid, WBC = white blood cell.

**Figure 1.**
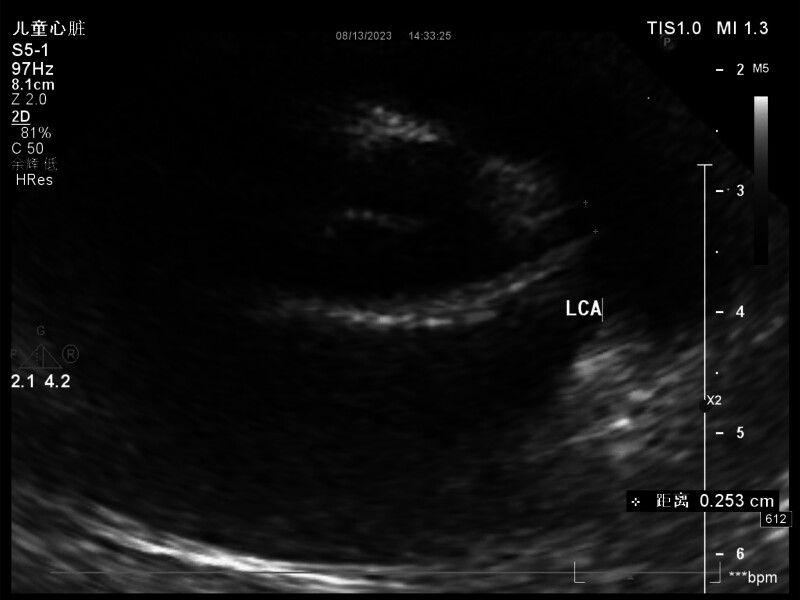
On day 4 of fever, the internal diameter of the beginning of the child’s left coronary artery (LCA) was approximately 2.5 mm, with a *Z* value of 2.06.

Physical examination revealed no abnormalities except for markedly swollen and erythematous hands and feet, a generalized erythematous rash, bilateral bulbar conjunctival hyperemia, chapped lips, strawberry tongue, and bilateral cervical lymphadenopathy, measuring approximately 2.5 cm in diameter, with a medium consistency and no adhesion to surrounding tissues. The second blood test results indicated elevated liver enzymes markers, significantly increased CRP levels, and an elevated erythrocyte sedimentation rate, leading to a clinical consideration of KD. Clopidogrel (1 mg/kg/d) was administered orally, and IVIG (2 g/kg) was administered intravenously. Echocardiography performed the following day revealed a *Z*-score of 2.06 for the left coronary artery, further supporting the diagnosis of KD (Fig. 1).

The child’s temperature remained elevated at 39.2°C after 36 hours of IVIG therapy, confirming a diagnosis of IVIG-resistant KD. Methylprednisolone was administered intravenously at a dose of 10 mg/kg every 8 hours for 3 days. Subsequently, the intravenous methylprednisolone dose was reduced to 2 mg/kg every 12 hours for 4 days, as the child’s temperature normalized, the rash improved, and the third blood test indicated normalization of liver enzymes and CRP levels. During the methylprednisolone taper, the child developed rhinorrhea, a mild cough, and a fever of 38.1°C. Respiratory nucleic acid testing was positive for rhinovirus and adenovirus, confirming coinfection with both viruses. The child’s temperature subsequently normalized. Following a repeat echocardiogram demonstrating normalization of the coronary arteries, intravenous methylprednisolone was continued at a reduced dose of 1 mg/kg every 12 hours for 2 days, followed by 1 mg/kg once daily for 1 day. Subsequently, methylprednisolone was transitioned to oral administration, with the dose reduced to 5 mg every 12 hours for 2 days, followed by 5 mg once daily for an additional 2 days.

**Figure 2.**
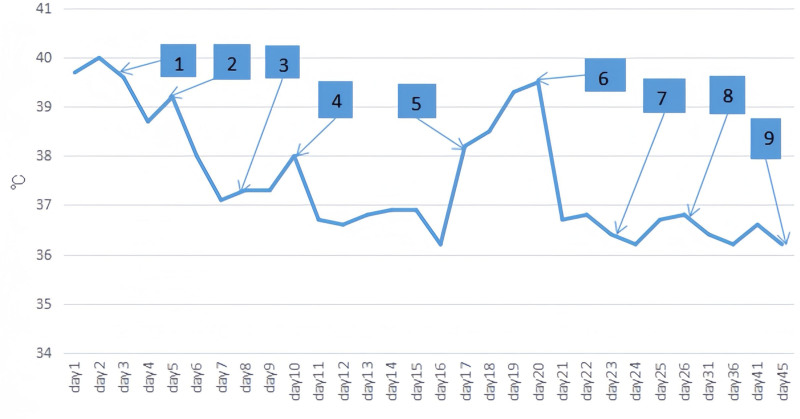
Trends of temperature of the patient during hospitalization. (1) Intravenous immunoglobulin and clopidogrel treatment. (2) High dosages of methylprednisolone treatment. (3) Reduce the methylprednisolone dosage gradually. (4) Both adenovirus and rhinovirus infected. (5) When the patient’s daily dosage of methylprednisolone tablets was lowered to one pill, fever returned. (6) Once more, intravenous methylprednisolone therapy was initiated once every 12 hours at a dose of 2 mg/kg. (7) The daily dose of methylprednisolone intravenous treatment was lowered to 2 mg/kg. (8) Start taking methylprednisolone tablets orally. (9) Stop taking methylprednisolone tablets.

On the day the methylprednisolone dose was reduced to 5 mg daily, the child developed a recurrent fever, with a peak temperature of 39.2°C. The child also exhibited erythema and swelling of the hands and feet, with no additional symptoms. Blood tests revealed an elevated CRP level of 58 mg/L, suggesting a potential recurrence of KD. Trends in the patient’s temperature during hospitalization are illustrated in Figure 2.

**Figure 3.**
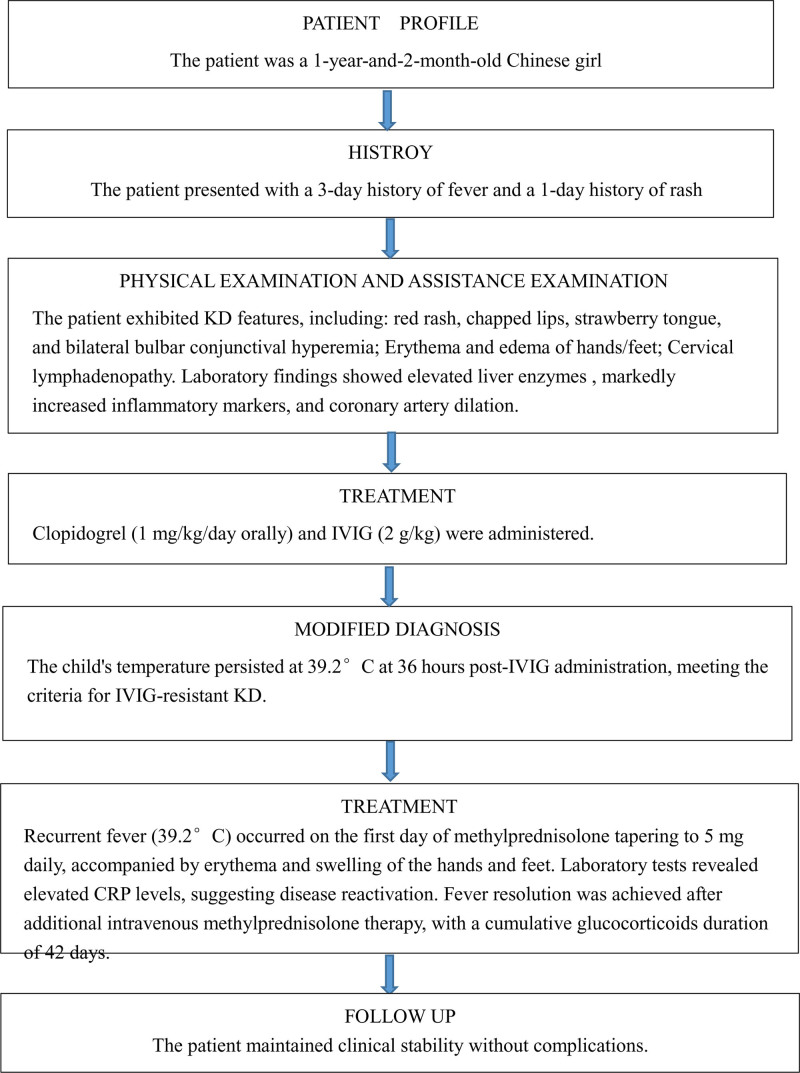
Timeline of the patient’s clinical course: presentation, diagnosis, treatment, revised diagnosis, and follow-up. CRP = C-reactive protein, IVIG = intravenous immunoglobulin, KD = Kawasaki disease.

The child’s temperature normalized following an additional course of intravenous methylprednisolone, administered at 2 mg/kg every 12 hours for 3 days, followed by 2 mg/kg once daily for an additional 3 days. CRP levels normalized, the erythema and swelling of the hands and feet resolved, and methylprednisolone was transitioned to oral administration, with the dose tapered by 5 mg every 5 days until discontinuation. Glucocorticoids was administered for a total of 42 days, and the patient was discharged on day 24 of hospitalization. Clopidogrel was discontinued after 6 weeks. Follow-up evaluations at 1 month, 3 months, 6 months, and 1-year post-discharge demonstrated normal echocardiograms, ECGs, and physical examinations. Figure 3 illustrates the timeline of the patient’s clinical course, from initial presentation to follow-up.

## 3. Discussion

Regarding the risk factors for IVIG-resistant KD, we reviewed relevant literature, and a summary is presented in Table 2. The findings of this study are consistent with the widely accepted view that children with KD exhibiting elevated levels of neutrophils (%), CRP, total bilirubin, aspartate aminotransferase (AST), and hyponatremia are more likely to be IVIG-resistant, as summarized in Table 2.

**Table 2:** Risk factors for predicting resistance to IVIG.

Year	Province, country	Cases	Risk factor for predicting IVIG-resistant
2023^[3]^	Sichuan of China	66	Longer length of hospitalization; older age; the N%, CRP, and CRP/albumin were significantly higher; L% and albumin were significantly lower.
2022^[4]^	Guangxi of China	63	The scoring system scores and the proportion of days of illness at primary treatment were higher; higher values of ALT, AST, and B/A ratio and lower values of the WBC and serum sodium.
2024^[5]^	Kedah of Malaysia	25	N% ≥60 %, CRP ≥ 80 mg/L, and male sex, while total bilirubin ≥ 9.4 μmol/L
2023^[6]^	Guangxi of China	38	Higher PCT,serum ferritin, serum soluble interleukin-2 receptor, ALT, total bilirubin, direct bilirubin, CRP, WBC, and N%. Significantly lower T-lymphocyte count, CD4 + T-lymphocyte count, albumin, sodium, Hb and PLT.
Current case	Zhejiang of China	1	The scoring system scores at primary treatment were higher; higher N%, CRP, AST, and total bilirubin; lower values of the serum sodium.

Abbreviations: ALT = serum alanine aminotransferase, AST = aspartate aminotransferas, B/A ratio = bilirubin-to-albumin ratio, CRP = C-reactive protein, Hb = hemoglobin, IVIG = intravenous immunoglobulin, L% = percentage of lymphocytes, N% = percentage of neutrophils, PCT = procalcitonin, PLT = platelets, WBC = white blood cell.

Regarding the association between IVIG resistance and WBC count in KD, the reference ^[4]^ suggests that IVIG resistance is more likely in patients with low WBC counts, while the reference ^[6]^ suggests the opposite; our findings are consistent with the study by the reference. ^[4]^ The Kobayashi score and 5 Chinese scoring systems were discussed in the study by the reference ^[4]^; the IVIG resistance data from this case were used for validation. The second Chinese scoring system in the study by the reference ^[4]^ and the scoring system in a study by the reference ^[5]^ demonstrated the highest predictive accuracy and reliability, whereas the third Chinese scoring system in the study by the reference ^[4]^ yielded contradictory results. When developing treatment plans for children with KD, clinicians may consider applying multiple scoring systems and adjusting their therapeutic strategies based on the outcomes.

According to current global guidelines for KD, IVIG therapy is the only recommended first-line treatment, with glucocorticoids not recommended as first-line therapy for all KD patients.^[7]^ Clinical practices and local recommendations vary significantly for IVIG-resistant patients, with a second dose of IVIG being more commonly administered than glucocorticoids.^[2]^ In recent years, a growing body of evidence has demonstrated that glucocorticoids are more beneficial than harmful for KD patients. A subgroup analysis indicated that glucocorticoids as first-line therapy reduce the incidence of coronary artery abnormalities.^[8]^ Broderick et al reviewed studies comparing IVIG with prednisolone and found no significant differences in side effects, acute coronary syndromes, or mortality between the 2 groups.^[9]^ Furthermore, glucocorticoids are associated with significantly lower costs compared to IVIG. This case provides valuable insights into the efficacy and safety of glucocorticoids in managing IVIG-resistant KD patients. The potential use of glucocorticoids as first-line therapy for KD patients with IVIG intolerance or IVIG resistance remains under investigation. High-quality studies are needed to accumulate further evidence over time, providing a stronger foundation for future recommendations.

According to current guidelines, glucocorticoids therapy for IVIG-resistant patients includes a single pulsed dose of intravenous methylprednisolone and a prolonged tapering course of prednisolone, with protocols varying significantly across countries: Denmark uses methylprednisolone (10–30 mg/kg/d), Japan employs intravenous prednisolone (2 mg/kg/d, tapered after CRP normalization) or methylprednisolone (30 mg/kg/d for 1–3 days), and Sweden utilizes intravenous methylprednisolone (6–30 mg/kg/d divided into 3 doses for 3 days) followed by oral prednisolone (1–2 mg/kg/d, tapered over 3 weeks).^[7]^ In China, the protocol involves prednisone (1–2 mg/kg/d), administered once daily in the morning, with a maximum dose of 60 mg/d or methylprednisolone (1–2 mg/kg/d), administered intravenously once or twice daily, with the dose tapered over 15 days following normalization of body temperature and CRP levels, gradually reducing from 1 to 2 mg/(kg/d) for 5 days, to 0.5 to 1 mg/(kg/d) for 5 days, and finally to 0.25 to 0.5 mg/(kg/d) for 5 days.^[10]^ In this case, the child received intravenous methylprednisolone and prednisolone for 13 days without interruption but subsequently developed recurrent fever, indicating a relapse of KD. The child achieved remission after 6 additional days of intravenous methylprednisolone, followed by a gradual taper over a 42-day period. This case suggests that rapid tapering of glucocorticoid therapy in IVIG-resistant KD patients may lead to treatment failure. While the guidelines recommend a 2 to 3 week course of glucocorticoids,^[7]^ we propose that individualized tapering regimens may be necessary for some patients. Our findings may assist in managing IVIG-resistant KD patients by offering insights into optimal glucocorticoid tapering strategies.

In addition to IVIG and glucocorticoids, other treatments for KD, which is associated with coronary artery aneurysms, include ulinastatin, infliximab, cyclosporine A, and plasma exchange. ^[11]^ However, the safety and efficacy of these treatments require further validation. These therapies may play a more prominent role in future updates to KD treatment guidelines.

## 4. Conclusions

In the management of IVIG-resistant KD, the most common approach is administering a second dose of IVIG, which is significantly more expensive than glucocorticoids. In this case, glucocorticoids were used as the first-line treatment, deviating from the standard approach. The patient achieved complete recovery following an adjusted strategy involving a prolonged glucocorticoid taper, despite an initial relapse due to an overly rapid reduction in steroid dosage. Notably, the patient experienced no adverse effects during the 42-day glucocorticoid regimen or subsequent follow-up. This case highlights the potential of glucocorticoids as a primary treatment option for IVIG-resistant KD. However, widespread adoption of glucocorticoids as first-line therapy requires robust clinical research to validate their efficacy and safety.

## Author contributions

**Writing – original draft:** Yifan Ren.

**Writing – review & editing:** Yifan Ren.
